# Risk for surgical complications after previous stereotactic body radiotherapy of the spine

**DOI:** 10.1186/s13014-017-0887-8

**Published:** 2017-09-11

**Authors:** Johannes Roesch, John B.C. Cho, Daniel K. Fahim, Peter C. Gerszten, John C. Flickinger, Inga S. Grills, Maha Jawad, Ronald Kersh, Daniel Letourneau, Frederick Mantel, Arjun Sahgal, John H. Shin, Brian Winey, Matthias Guckenberger

**Affiliations:** 1 Department of Radiation Oncology, University Hospital Zurich, Zurich, Switzerland; 2 Princess Margaret Cancer Centre, Radiation Medicine Program, Toronto, Canada; 3 Department of Neurosurgery, William Beaumont Hospital, Royal Oak, Michigan USA; 4 Department of Neurological Surgery, University of Pittsburgh Medical Center, Pittsburgh, Pennsylvania USA; 5 Department of Radiation Oncology, University of Pittsburgh Medical Center, Pittsburgh, Pennsylvania USA; 6 Department of Radiation Oncology, William Beaumont Hospital, Royal Oak, Michigan USA; 7 Department of Radiation Oncology, Riverside Medical Center, Newport News, Virginia USA; 8 Department of Radiation Oncology, University Hospital Würzburg, Würzburg, Germany; 9 Department of Radiation Oncology, Sunnybrook Health Sciences Centre, Toronto, Canada; 10 Department of Neurosurgery, Massachusetts General Hospital, Boston, Massachusetts USA; 11 Department of Radiation Oncology, Massachusetts General Hospital, Boston, Massachusetts USA

**Keywords:** Spine tumor, Stereotactic radiotherapy of the spine, Radiosurgery, SBRT, Spine surgery, Complications

## Abstract

**Object:**

Stereotactic body radiotherapy (SBRT) for vertebral metastases has emerged as a promising technique, offering high rates of symptom relief and local control combined with low risk of toxicity. Nonetheless, local failure or vertebral instability may occur after spine SBRT, generating the need for subsequent surgery in the irradiated region. This study evaluated whether there is an increased incidence of surgical complications in patients previously treated with SBRT at the index level.

**Methods:**

Based upon a retrospective international database of 704 cases treated with SBRT for vertebral metastases, 30 patients treated at 6 different institutions were identified who underwent surgery in a region previously treated with SBRT.

**Results:**

Thirty patients, median age 59 years (range 27–84 years) underwent SBRT for 32 vertebral metastases followed by surgery at the same vertebra. Median follow-up time from SBRT was 17 months. In 17 cases, conventional radiotherapy had been delivered prior to SBRT at a median dose of 30 Gy in median 10 fractions. SBRT was administered with a median prescription dose of 19.3 Gy (range 15–65 Gy) delivered in median 1 fraction (range 1–17) (median EQD2/10 = 44 Gy). The median time interval between SBRT and surgical salvage therapy was 6 months (range 1–39 months). Reasons for subsequent surgery were pain (*n* = 28), neurological deterioration (*n* = 15) or fracture of the vertebral body (*n* = 13). Open surgical decompression (*n* = 24) and/or stabilization (*n* = 18) were most frequently performed; Five patients (6 vertebrae) were treated without complications with vertebroplasty only. Increased fibrosis complicating the surgical procedure was explicitly stated in one surgical report. Two durotomies occurred which were closed during the operation, associated with a neurological deficit in one patient. Median blood loss was 500 ml, but five patients had a blood loss of more than 1 l during the procedure. Delayed wound healing was reported in two cases. One patient died within 30 days of the operation.

**Conclusion:**

In this series of surgical interventions following spine SBRT, the overall complication rate was 19%, which appears comparable to primary surgery without previous SBRT. Prior spine SBRT does not appear to significantly increase the risk of intra- and post-surgical complications.

## Background

The skeleton is one of the most common sites of metastatic disease spread in cancer patients with approximately 300,000 cases per year in the United States [1, 2]. Depending on the type of cancer, bony metastases occur in up to 70% of patients [3]. Furthermore, the spine is the most common site of bony metastasis, with postmortem detection of vertebral metastases in 30 to 36% of patients who died from neoplastic disease [4–6].

Pain medication combined with conventional radiotherapy (RT) is the standard of care for symptomatic bone metastases. However, recent developments in RT treatment planning and delivery enabled dose escalation within one or few treatment sessions and thus stereotactic body radiation therapy (SBRT) has emerged as a promising treatment technique [7]. By offering high rates of symptom relief and local control combined with low rates of toxicity, its acceptance and use are increasing [8, 9]. Nonetheless, symptomatic local failure or spinal instability after spine SBRT may occur, generating the need for subsequent surgery in the irradiated region [10, 11].

Prior RT is known to cause tissue alterations which might lead to difficult surgical conditions and subsequently to an increased risk of surgical complications. On the contrary, single dose RT might also be used to confine surgical induced fibrosis, which is common practice in keloid surgery, but was also found beneficial to prevent peridural fibrosis [12]. Surgical risk profiles following SBRT have been described in case series for recurrent tumors inside the lung [13, 14]. Despite fibrotic changes within the irradiated area, no increased surgical toxicity due to previous SBRT was reported for these cases. This study was conducted to specifically evaluate whether there is an increased risk of intra- and post-surgical complications after spine SBRT compared to historical results of surgery without previous SBRT.

## Methods

Based on a retrospective international database of 704 patients treated with SBRT for vertebral metastases at 7 centers, 30 patients were identified who underwent surgery in a region previously treated with SBRT. Data for 32 surgical procedures, their treatment characteristics and complications were available, covering a period from November 2006 to July 2013. All centers are members of the Elekta Spine Radiosurgery Research Consortium and ethics approval was obtained by all institutions.

For each case the original surgical report was evaluated regarding the localization, reason and type of surgery, as well as intraoperative complications such as increased tissue fibrosis or blood loss. Additionally, patient charts were screened for the following postoperative complications: delayed wound healing (surgical site infection and/or wound dehiscence), blood loss, spinal instability, increased neurological deficit, pain, pneumonia, deep vein thrombosis, pulmonary embolism, and death within 30 days after surgery.

For plotting and statistical analyzes Microsoft Excel 2016 and R Studio 0.999 were used and next to descriptive statistical figures, Shapiro Wilk-, Wilcoxon rank sum-, Chi-Square- and Fishers exact test were applied. To account for multiple testing *p*-values were adjusted according to the false discovery rate approach [15].

## Results

Thirty patients with a median age of 59 years (range 27–84 years) underwent SBRT for 32 vertebral metastases followed by surgery in the same region. Metastases of breast cancer and kidney cancer accounted for more than half of the cases. Median follow-up was 17 months from date of SBRT and 8 months from date of surgery. SBRT as well as conventional RT was exclusively delivered with Linear accelerators by Elekta. Doses for conventional RT were prescribed to the 95%-isodose according to ICRU 50/62. In constrast SBRT doses were prescribed in-homogeneously to the PTV encompassing isodose with variable maximum doses according to center and treating physician. In 17 cases, conventional radiotherapy had been delivered prior to SBRT at a median dose of 30 Gy in median 10 fractions. SBRT was most frequently administered in a single fraction with a median prescription dose of 18.4 Gy (median EQD2/10 = 43.5 Gy). Fractionated SBRT used a median of three fractions and a total dose of median 24 Gy (median EQD2/10 = 44 Gy). Overall, the median prescribed physical and EQD2/10 doses were 19.3 Gy and 44 Gy, respectively. Split into two groups of primary SBRT and secondary SBRT, the latter with a history of conventional RT in the same region, no significant difference was seen according to patient characteristics, SBRT dose and fractionation or surgical features. For a detailed assembly of fractionation and dose schemes the reader is referred to Tables 1 and 2.

**Table 1:** Patient and disease characteristics

Patient characteristics	*N* = 30	
Median age at Operation	59y (27–84)	
Gender	Females: 17	Males: 13
Primary site	Number	Percentage
Kidney	10	33%
Breast	7	23%
Prostate	3	10%
Melanoma	2	7%
NSCLC	1	3%
Colorectal	1	3%
Other	6	20%

**Table 2:** Available RT-characteristics of all SBRT, primary SBRT only, secondary SBRT only with a history of conventional RT in the same region and conventional RT prior to SBRT

	SBRT over all		Primary SBRT		Secondary SBRT		Prior conventional RT	
	Number of treatments	Median prescription dose (range)	Number of treatments	Median prescription dose (range)	Number of treatments	Median prescription dose (range)	Number of treatments	Median prescription dose (range)
Over all	32	19,3 (15–65)	15	21.7 Gy (15.7–65 Gy)	17	18 Gy (15–24 Gy)	16	30 Gy (20–40 Gy)
1 fraction	25	18,4 Gy (15–24 Gy)	10	20.5 Gy (15.7–24 Gy)	15	18 Gy (15–20 Gy)	3	20 Gy in 5 fractions
2 fractions	3	24 Gy	2	24 Gy	1	24 Gy	11	30 Gy in 10 fractions
3 fractions	2	17,7 Gy (15–20,4 Gy)	1	20.4 Gy	1	15 Gy	1	35 Gy in 14 fractions
5 fractions	1	25 Gy	1	25 Gy			1	40 Gy in 20 fractions
17 fractions	1	65 Gy	1	65 Gy				

**Fig. 1:**
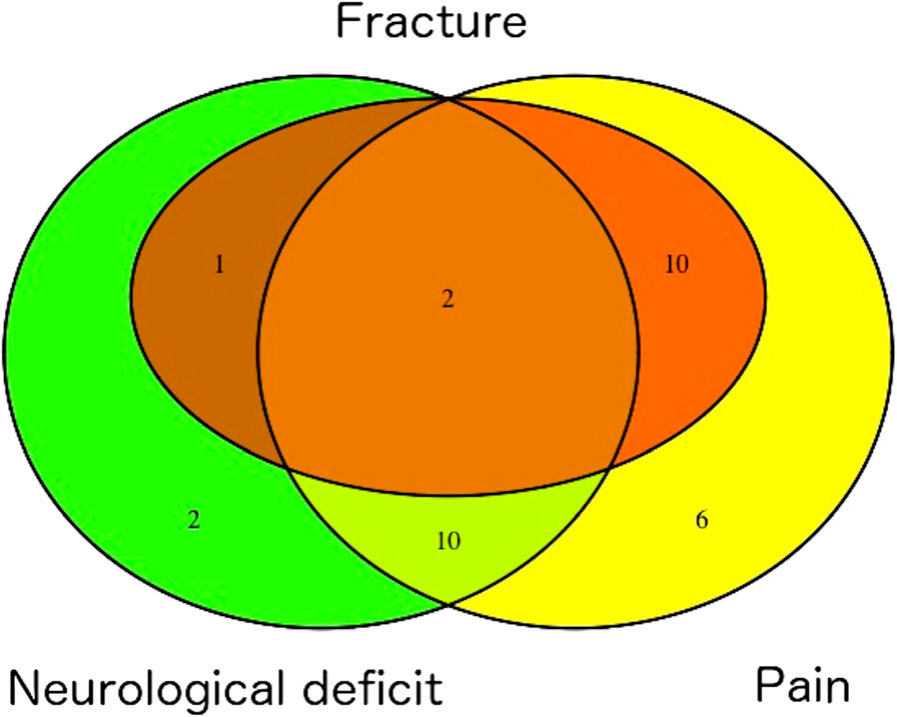
Venn diagram illustrating the reason for surgery: fracture = red circle, neurological deficit = green circle, pain = yellow circle

**Fig. 2:**
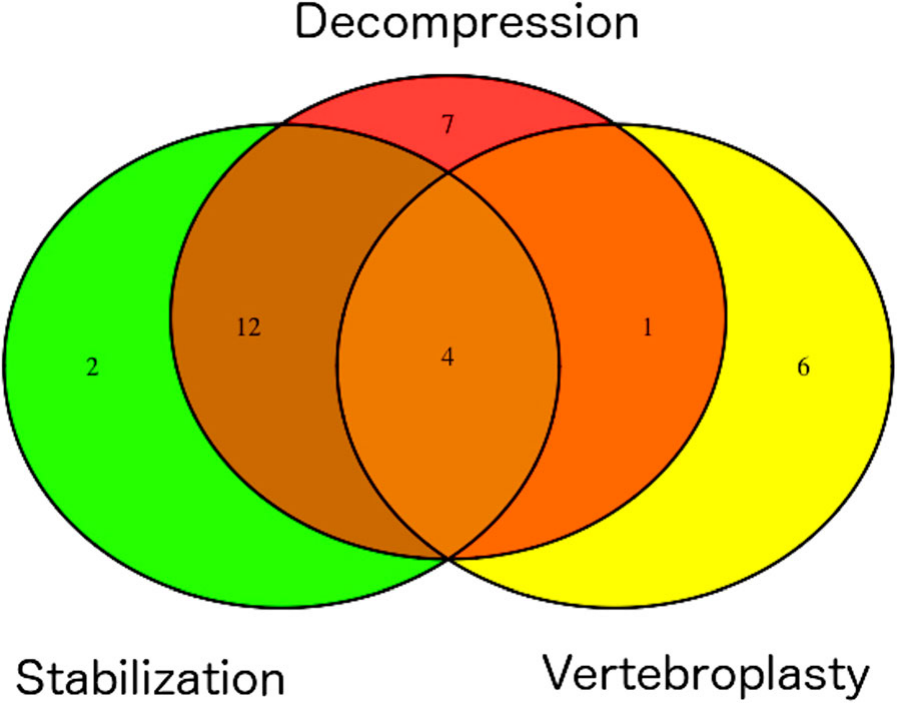
Venn diagram illustrating the type of surgery: decompression = red circle, stabilization = green circle, vertebroplasty = yellow circle

The median time between SBRT and surgical salvage therapy was 6 months (range 1–39 months). The most frequent reason for surgery was progressive pain (*n* = 28) followed by progressive neurological deterioration (*n* = 15), fracture of the vertebral body (*n* = 13) or in one case spondylolisthesis (see Fig. 1). Open surgical decompression (*n* = 24) and/or stabilization using pedicle screws and rod fixation (*n* = 18) were the most frequently performed surgical procedures. Vertebroplasty / balloon kyphoplasty was performed 11 times in 10 patients, 5 of which in a combined approach with decompression and placement of spinal instrumentation devices (see Fig. 2).

Due to different risks for vertebroplasty alone and more complex surgery the patient population was divided into two groups for risk analyzes.

Five patients were treated with vertebroplasty only and formed the low-risk group. One patient was treated with SBRT to T10 and T12 followed by simultaneous vertebroplasty at both sites 10 months later due to increased pain and progressive vertebral body compression demonstrated on imaging without complication. SBRT prescription doses in this subgroup ranged from 15 to 24 Gy with 20.5 Gy in median in 1–3 fractions (median EQD2/10 = 50 Gy). Two patients had conventional RT prior to SBRT (40 Gy in 20 fr. / 30 Gy in 10 fr.). Overall, none of these five patients suffered from any complication intra- or postoperatively except residual pain in two cases.

Twenty-five patients in the high-risk group received 26 surgical salvage treatments. SBRT was delivered to a median prescription dose of 18.4 Gy (15–65 Gy) in mostly one fraction (1–17 fractions). Fifteen patients had received conventional RT prior to SBRT: Eleven patients had received 30 Gy in 10 fractions, three patients had received 20 Gy in 5 fractions and one patient had received 35 Gy in 14 fractions. One patient was sequentially treated in the L4 region starting with conventional irradiation (30 Gy in 10 fractions) followed by SBRT (19.4 Gy single fraction, time interval of 11 months), surgical decompression (time interval of 5 months), SBRT (17.6 Gy single fraction, time interval of 2 months) and subsequent repeat surgical decompression (time interval 8 months) without complications.

Intraoperatively, increased fibrosis complicating the surgical procedure was explicitly stated in one out of 26 surgical reports leading to an unintentional durotomy without further complications in this particular case. A second patient with intraoperative durotomy experienced an increased neurological deficit and delayed wound healing after surgery. In both cases the durotomy was sufficiently repaired during the surgical procedure and no revision surgery was needed.

Information regarding blood loss during surgery was available for 18 interventions. The median blood loss for cases was 500 ml (range 5–2000 ml). Five patients experienced a blood loss of greater than 1 l during the procedure; in all five cases, an open decompressive surgery in combination with stabilization devices was performed. Two of these five patients had delayed wound healing, and one patient died shortly after surgery. Furthermore, four of these five patients had received prior conventional fractionated RT in addition to SBRT at the treated level. The finding of local complications occurring only in the group of patients with conventional RT in addition to SBRT was not statistically significant. No significant differences were seen in this subgroup of patients in terms of primary site of disease, SBRT prescription dose, and time interval between SBRT and subsequent surgery.

Postoperatively, seven complications occurred which are summarized in Table 3. Overall, two cases of delayed wound healing were observed. One of these cases also suffered from an increased postoperative neurological deficit as already described. There was no case of subsequent spinal instability after surgery. Residual pain in the surgical area was observed in eight patients. Three patients had documentation of systemic complications: one pneumonia, one urinary tract infection and deep vein thrombosis, and one patient died shortly after the surgical procedure due to causes that could not be determined.

**Table 3:** Systemic, local and overall complication rate for 26 surgical interventions (open surgical decompression and/or instrumental stabilization) at 25 sites in 25 patients with previous conventional RT and SBRT or SBRT only at the same level

	Overall (*n* = 26)	RT + SBRT (n = 15)	SBRT (*n* = 11)
Systemic complications	4 (in 3 patients = 12%)	1 (7%)	3 (in 2 patients = 27%)
Pneumonia / urinary tract infection	1 / 1	0	1 / 1
Deep vein thrombosis	1	0	1
Perioperative death (within 30 days)	1	1	0
Local complications	3 (in 2 patients = 8%)	3 (in 2 patients = 13%)	0
Increased neurological deficit	1	1	0
Spinal instability	0	0	0
Delayed wound healing	2	2	0
Postoperative bleeding/Hematoma	0	0	0
Overall complication rate	7 (in 5 patients = 19%)	4 (in 3 patients = 17%)	3 (in 2 patients = 27%)

## Discussion

This study evaluated the risks of in-field spine surgery subsequent to SBRT. It is well documented that the overall complication rate associated with palliative spinal surgery in oncological patients is high. Due to their malignant disease and often multiple local and systemic therapies, cancer patients frequently suffer from multiple risk factors for both local as well as systemic intraoperative and postoperative complications. In the current study, we observed an overall surgical complication rate of 19% for the high risk group. Despite heterogeneous definitions and grouping of complications among previously published reports, this risk does not appear to be increased compared to series of surgical complications in comparable patients who undergo surgery without prior SBRT [16–19]. Regarding systemic complications, our rates of pneumonia (4%) and deep vein thrombosis (4%) are also within the expected range. In this cohort, one patient died within 30 days following surgery (4%) and similar mortality rates ranging from 3% to 13% have been reported in several retrospective and prospective surgical series [16–18, 20–23].

Regarding local complications, general risk factors such as age, stress, diabetes, medication, drugs, nutritional status or extent and duration of surgery have been described [24]. Explicitly in spinal surgery, a preoperative neurological deficit resulting from close proximity of the tumor to neural structures is a factor further increasing the risk for surgical complications [25, 26]. In a large retrospective study including almost 1000 patients by Finkelstein et al., a preoperative neurological deficit contributed to a 19% increase in mortality and a 71% increase in the risk of postoperative wound infection [16]. With 15 procedures undertaken due to progressive neurological deficit, patients with this risk factor were overrepresented in our study. However, postoperative complications were equally distributed amongst both of the subgroups.

Fibrotic tissue alteration after RT and SBRT is a well-known phenomenon. Therefore, this alteration might lead to difficult surgical conditions and to an increased risk of surgical complications. Surgical risk profiles following SBRT have been described in case series for recurrent tumors inside the lung [13, 14]. Despite fibrotic change within the irradiated area, no increased surgical morbidity due to previous SBRT was reported in these series of lung surgery. Regarding the time interval between irradiation and subsequent surgery, fibrotic remodeling inside the lung caused by SBRT seems to take place up to 1 year until reaching its maximum [27].

The evidence for increased fibrosis after spine SBRT is limited. By using radiosurgical doses of 10 Gy and 20 Gy in a mouse model, the extent and severity of epidural fibrosis after irradiation was shown to be dose and time dependent [28]. In our series only one surgical report explicitly mentioned complicating surgical conditions due to fibrotic remodeling. Additional cases with increased fibrosis but not complicating the surgical procedure may be assumed, but were not stated. In this context, two cases of durotomy and intraoperative cerebrospinal fluid leakage were observed which corresponds to a crude rate of 6.3% in 32 surgical sites. Durotomy may be associated with increased perioperative morbidity and long-term neurological sequelae [29]. According to large single institution series accidental durotomy occurs in 3.6–7.6% in primary spine surgery but its incidence increases in lumbar location or patients with pre-existing fibrosis, which is best validated for revision surgery [30–34]. For total *en bloc* spondylectomy, preoperative conventional RT was a dose and time dependent risk factor for durotomy [35]. The authors reported an increased complication rate above 40 Gy or after a time interval of 1 year between RT and surgery.

Intraoperative blood loss correlates with the extent of surgery, leads to increased stress of the cardiovascular system and might require blood transfusions. Furthermore, it is associated with an increased wound infection rate [36]. In general, blood loss exceeding 2000 ml is uncommon in spinal surgery [33]. In our patient cohort we observed a median blood loss of 500 ml (range 5–2000 ml). Five patients lost more than 1 l of blood during the operation. This large variability in blood loss is most likely explained by the variety of surgical interventions performed in our study ranging from percutaneous instrumental stabilization to wider tumor resections partly combined with stabilization. Shehadi et al. reported a median blood loss of 500 ml in anterior or posterior approach only, 1350 ml for the combined simultaneously, and 2500 ml for the combined staged approach [37]. Similarly, Quan et al. reported a mean blood loss of 718 ml for surgeries with both anterior and posterior approaches [21]. In the series reported by Demura et al., a median blood loss of 1560 ml was seen, but only one quarter of patients received palliative surgery. In the majority of patients *en bloc* spondylectomy or vast debulking was performed. Likewise, Wang et al. reported a median blood loss of 1500 ml during their interventions where the spinal cord was circumferentially decompressed by a posterolateral transpedicular approach [38].

Conventional RT is a known risk factor for postoperative wound healing complications, like wound dehiscence or infection, in different organ sites including the spine [26, 39, 40]. Several large retrospective series reported increased rates of surgical site infections of 12–40% in pre-irradiated tissue compared to only 1–15% without prior RT [21, 25, 41–44]. Another smaller series of 42 patients reported two wound breakdowns after revision surgery and one wound infection in pre-irradiated patients [45]. However, one must be aware of a possible negative selection bias and poor description of RT regarding dose and interval between surgery and RT within this retrospective data. On the contrary, some studies could not confirm this relationship. Shehadi et al. retrospectively and Wang et al. prospectively could not find an association between preoperative irradiation and postoperative wound infection [37, 38]. In the latter, 60% of 140 patients were irradiated before surgery with mostly 30 Gy in 10 fractions and an interval between RT and surgery of more than 30 days. Table 4 summarizes studies analyzing surgical complications after spine surgery.

**Table 4:** Summary of trials with general and local complication rates after spine surgery with and without previous RT

Study, year	Main type of surgery	Complications rate			Wound complications			Perioperative deaths = 30 days mortality
		Overall / Number of patients	RT + S / number of patients	S alone / number of patients	overall / number of surgeries	RT + S / number of surgeries	S alone / number of surgeries	
Demura, 2009 [41]	Debulking / stabilization	–	–	–	7.1% / 113	31.8% / 22	1.1% / 91	–
Ghogawala, 2001 [43]	Posterolateral decompression and stabilization	/ 85	–	–	–	32% / 28	12% / 34	–
Lau, 2013 [34]	–	21.7% / 106	23.5% / 81	16% / 25	3.8%/106	–	–	1 (0.9%)
Pascal, 1998 [25]	Post + ant. approach	18.6% / 145	–	–	11% / 145	12%	1%	3 (2%)
Quan, 2011 [21]	Post + ant. approach	26% / 118	42% / 19	–	6.8% / 118	15.8% / 19	5% / 99	9 (7.6%)
Shehadi, 2007 [37]	–	–	–	39% / 87	–	No significant difference		–
Sundaresan, 2002 [42]	Ant. Approach + post. stab.	80	15% / 40	40% / 40	13.8% / 80	25% / 40	2.5% / 40	1 (1.3%)
Wang, 2004 [38]	Posterior approach	14.3% / 140	84	56	11.4% / 140	No significant difference		6 (4.3%)
Wise, 1999 [26]	Post + ant. approach	25% / 80	15.5% / 41	9.3% /39	17.1% / 80	–	–	2 (2.3%)
Yokogawa, 2014 [35]	Total en bloc spondylectomy	40% / 50	77.8% / 18	18.8% / 32	8% / 50	22.2% / 18	0% / 32	–

Whether there is a dose-effect relationship for surgical complications following radiotherapy remains unclear. Most studies reporting post-surgical wound healing issues described characteristics of radiotherapy only poorly. On the other hand, studies with an emphasis on radiation oncology often do not report surgical outcome or complications. Nonetheless, there is some clinical and preclinical data suggesting a dependency between RT-dose and postoperative wound complication rate [46]. Further conclusions by analogy can be drawn from other treatment sites. Single fraction doses of up to 7 Gy are frequently and safely administered immediately prior to surgery to reduce the risk for heterotopic ossification [47, 48]. Complication rates increase after preoperative conventionally fractionated radiotherapy of 50 Gy and further increase with an additional boost in sarcoma treatment [49]. Likewise, Yokogawa et al. reported a tendency for more frequent wound complications after spinal surgery for patients pretreated with >40 Gy [35]. The interval between surgery and RT might also be an important risk factor for post-surgical wound healing complications. Irradiation interferes with immediate wound healing in the short term and favors fibrotic tissue alterations in the long term. To minimize the risk of radiotherapy-induced wound complications, recommendations regarding the right point in time for surgery following RT range between an absolute minimum of one to 3 weeks [43, 46, 50]. However, such planning is not always possible in the situation of the need for urgent surgery.

The issue of wound complications in patients who underwent spine surgery for metastasis with a history of prior radiotherapy was studied by Keam et al. [51]. In their series of 165 patients 35 patients received hypofractionated RT with doses of 18–30 Gy in 1–5 fractions. The 6-month cumulative incidence of wound complications for conventional RT was 17% and for hypofractionated RT was 6%. There was no significant difference, but a tendency for higher doses per fraction being associated with lower wound complication rates.

In our setting of surgery after SBRT, we observed delayed wound healing in two cases, corresponding to a crude rate of 8%, which is in the range of large surgical spine series of 7–12% [16, 21, 25, 38]. Furthermore, local complications were only seen in patients with prior history of conventional RT in addition to stereotactic RT supporting the thesis of Keam et al. A possible explanation for lower risk after SBRT despite higher biologically effective radiation doses compared to conventional palliative radiotherapy may be given by a more conformal dose distribution reducing the affected tissue to a minimum.

The favorable outcome in our study might be explained by the high level of radiotherapy and neurosurgical experience and expertise of all participating centers. All institutions have a clinical and scientific focus on the multi-disciplinary treatment of vertebral metastases. Additionally, SBRT and salvage surgery were performed at the same institution in all cases: this guarantees the best access of the neurosurgeon to all the relevant information of the previous SBRT treatment which might be beneficial for patient selection and planning of the surgical procedure. Still, expected complications were seen in this study. Bearing this in mind our results may not be assignable to centers with less expertise.

The relative small number of highly selected cases and its retrospective type are major limitations of this study. Therefore, statistical analyzes are to be treated with caution. Heterogeneity and missing details of surgical procedures like blood loss reflects the data’s origin from a multicenter database. Furthermore, heterogeneous definitions of complications among different studies may impede an actual comparison.

## Conclusion

Based on the results of this multi-institutional study, surgery in a specialized center appears to be a safe salvage option in case of symptomatic local tumor recurrence or spinal instability after spine SBRT. The rate of complications does not appear to be higher compared to series without prior SBRT or series with prior conventional radiotherapy.

## Abbreviations

RT: Radiation therapy
SBRT: Stereotactic body radiation therapy
